# Numerical Study on Effect of Contact and Interfacial Resistance on Thermal Conductivity of Dispersed Composites

**DOI:** 10.3390/ma16020517

**Published:** 2023-01-05

**Authors:** Atsushi Kondo, Hiroshi Matsuura, Yoshiharu Ito

**Affiliations:** 1 Department of Mechanical Engineering, Nippon Institute of Technology, 4-1 Gakuendai, Miyashiro-machi, Minamisaitama-gun 345-8501, Saitama Prefecture, Japan; 2 Department of Mechanical Systems Engineering, Aichi University of Technology, 50-2 Manori, Gamagori 443-0047, Aichi Prefecture, Japan

**Keywords:** composites, interfacial thermal resistance, thermal conductivity

## Abstract

A series of finite element analyses were conducted to clarify the effect of contact and interfacial resistance between constituents on effective thermal conductivities of dispersed composites. Equally dispersed fillers in FCC (face-centered cubic) and BCC (body-centered cubic) material systems were extracted from cyclic microstructures as unit cell models. In addition to spherical fillers, a polyhedron called the Wigner–Seitz cell that can realize a fully packed microstructure was chosen as the shape of the filler to investigate the effect of contact between the high volumetric fraction of fillers. The effective thermal conductivities of the resulting composites were calculated based on the FEA results and compared to the theoretical results for various volume fractions of the fillers including the maximum packing fraction. The following conclusions were obtained from the present study: 1. The effect of the contact depending on the shape and configuration of the fillers has more of a significant influence on the effective thermal conductivity than the influence of the increase in the volume fraction of the fillers. 2. When the contact occurred, the effective thermal conductivity became more than double that without contact. 3. Interfacial thermal resistance must be less than the order of 10^−4^ m^2^ K/W to obtain improvement in the effective thermal conductivity by compounding the fillers.

## 1. Introduction

Dispersed composites which enable high thermal conductivity have been attracting attention from a variety of industries such as the electronics industry where heat dissipation is crucial for the better performance of their products. Polymer compounds have suitability for conventional use as thermal conductive adhesives and electric cables [1,2] and also have the potential to replace metallic materials with applications such as electronic packaging materials [3]. Metallic matrix composites are chosen when their other superiorities such as low coefficient of thermal expansion are needed [4]. Variety forms of fillers including particles, fibers and lattices of nanomaterials such as graphene are adopted [5]. The characteristics of the composites are known to be influenced by a lot of factors such as shape and fraction of fillers and conditions of interface between constituents. Therefore, a lot of experiments are required to achieve the desired quality of the composites. In this context, methods contributing to a reduction in such experiments have been sought. Burger et al., (2016) provided a comprehensive review of the mechanisms of heat conduction in composites [6].

Theories for the prediction of effective thermal conductivity of dispersed composites have been proposed in earlier studies [7,8,9]. Maxwell (1904) derived the following equation by solving the differential equation of thermal conduction in an infinite medium with a spherical filler in the center based on the assumption that temperature is constant at infinity [7].

(1) $$K_{c}=\frac{K_{f}+2-2v_{f}\left(1-K_{f}\right)}{K_{f}+2+v_{f}\left(1-K_{f}\right)}$$

where *K_c_* and *K_f_* express the ratio of thermal conductivity of the resulting composites to that of the matrix material, and the ratio of thermal conductivity of fillers to that of the matrix, respectively. *v_f_* is the volume fraction of fillers. When the volume fraction is high, the effective thermal conductivity derived from this equation is significantly different to that which is experimentally obtained because the thermal interaction between neighboring fillers is not taken into account in this theory. Bruggeman (1935) approximately introduced the thermal interaction between fillers by recursively adding an inclusion to composites solved by the way of Maxwell’s theory and obtained using the following equation [8].

(2) $$1-v_{f}=\frac{K_{c}-K_{f}}{K_{c}{}^{1/3}\left(1-K_{f}\right)}$$

Equations (1) and (2) are widely accepted because they form the upper and lower boundaries of most of the experimental data with spherical fillers, as reported by Meredith [9].

Since Bruggeman’s equation tends to overestimate the thermal interaction between fillers, Meredith (1959) expanded the theory by considering the interaction between pairs of the closest fillers and obtained the following equation which can be applied to cases with a relatively high volumetric fraction of fillers [9].

(3) $$K_{c}=\left[\frac{2+v_{f}\left(wK_{f}-1\right)}{2+v_{f}\left(w-1\right)}\right]\left[\frac{2\left(1-v_{f}\right)+v_{f}wK_{f}}{2\left(1-v_{f}\right)+v_{f}w}\right]$$

This equation is applicable to various shapes of inclusions by using the shape parameter *w*. In the case of spherical fillers, the shape parameter *w* is expressed as

(4) $$w=3/\left(K_{f}+2\right).$$

Theories with consideration for more complex phenomena such as thermal contact have been proposed recently, for example, by Bahrami (2006) [10].

**Figure 1:**
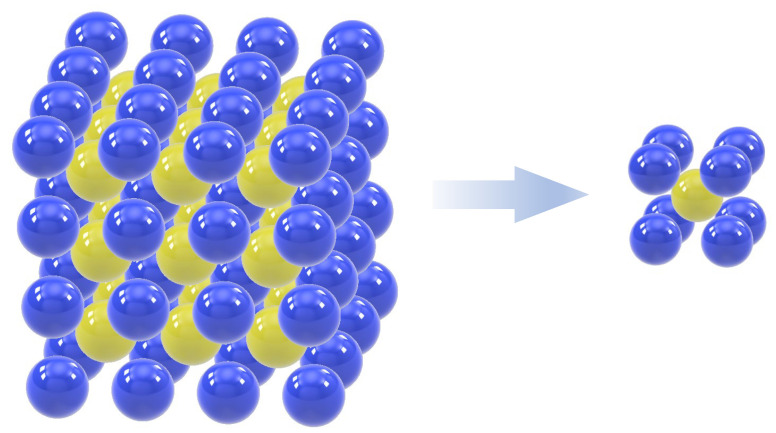
Extraction of a unit cell model from cyclic microstructures [14].

Although good agreement between the theoretical predictions and experiments has been made possible by the above-mentioned theories, adjustment of parameters based on a lot of experiments to compensate for the approximation error is still required for good prediction. Therefore, more rigorous treatment of the thermal interaction has been studied using numerical analyses. Kortschot and Woodhams (1988) successfully predicted the effective thermal conductivity of composites with randomly oriented fillers using a statistical computer simulation [11]. The finite element method is a superior method for detailed investigation of the physical phenomena such as heat transfer in the composite material because it directly incorporates the constitutive equation of the constituent materials of the composites into the analyses. Ramani and Vaidyanathan (1995) were two of the first scientists who adopted the finite element method to analyze heat transfer in the microstructures of the composites [12]. However, local thermal interaction such as contact and thermal interfacial resistance were not yet considered in their analyses. Buonano and Carotenuto (2000) considered contact between fillers in their finite element analyses [13], and Matt and Cruz (2007) proposed finite element formulation that accounted for interfacial thermal resistance [14]. Haddadi et al., (2013) applied the method for analysis of the idealized microstructures with hollow particles with consideration of interfacial thermal resistance [15]. A comprehensive review of theoretical and numerical methods to predict the effective thermal conductivity of composites was provided by Pietrak et al., (2015) [16]. In these studies, microstructures of the composites were assumed to have cyclic symmetricity. One of the cyclic microstructures was extracted as a model called a unit cell model, as shown in Figure 1, and was analyzed using boundary conditions according to the cyclic symmetricity [14]. This numerical method that links macroscopic and microscopic phenomena of the composite materials is called homogenization and applies to a wide range of materials [17,18,19,20].

A lot of studies reported that interfacial thermal resistance is one of the most important factors required to understand heat transfer between two materials [21,22,23,24,25]. The first experiment to evaluate interfacial thermal resistance was conducted for an interface between liquid helium and a solid by Kapitza (1941) [26] and later expanded to solid–solid interfaces by scientists such as Weis (1972) [27]. Khalatnikov (1959) proposed the first theory to predict interfacial thermal resistance called the acoustic mismatch model [28]. The acoustic mismatch model is based on the physics of phonon transport governed by continuum acoustics. It can be interpreted as the acoustic analog of Snell’s law for electromagnetic waves which describes the relationship between the angle of incidence and refraction. Although the prediction by the acoustic mismatch model well agreed with measurements of temperature lower than about 1 K, it was found that there was a slight difference between them when temperature was higher. In the acoustic mismatch model, no scattering is assumed to occur at the interface in spite of the fact that the effect of the scattering is not negligible in that temperature range. A model with consideration for the scattering called the phonon diffuse mismatch model was then proposed by Swartz and Pohl (1989) [29] and has widely been used recently, for example, in a report by Wang et al., (2007) [30].

These experimental and numerical works found that the effective thermal conductivity increases with fraction of fillers, and when the fraction exceeds a threshold called critical fraction, significant improvement in the thermal conductivity occurs. This phenomenon was called percolation, whose empirical law was proposed by Landauer (1978) [31]. However, its application to thermal conductivity still needs parameter fittings due to a lack of thorough understanding about the role of the local interaction such as contact and interfacial resistance.

In the present study, a series of finite element analyses were conducted with the aim of clarifying the influence of the above-mentioned local interaction on the improvement in effective thermal conductivity of composites as well as providing a guideline to fabricate better composite materials in the industry. First, finite element models of microstructures of composites with dispersed fillers with different shapes under consideration of contact and interfacial thermal resistance were developed. Then, the effect of the contact condition was investigated from detailed observation of the heat flux field obtained from the FEAs with various volume fractions of fillers including maximum packing fraction. Moreover, how interfacial thermal resistance operates within a practical range in the industry was investigated.

## 2. Numerical Analyses

### 2.1. Constitutive Equations

In the present study, thermal conduction on a macroscopic scale is assumed to be governed by Fourier’s law which can be written as

(5) q=−κ·∇T

where heat flux vector **q** is expressed in the following matrix form [32].

(6) $$q={\left[\begin{array}{ccc} q_{x} & q_{y} & q_{z} \end{array}\right]}^{T}$$

Matrix form of the gradient operator **∇** is

(7) $$\nabla ={\left[\begin{array}{ccc} \frac{d}{dx} & \frac{d}{dy} & \frac{d}{dz} \end{array}\right]}^{T}.$$

Thermal conductivity **κ** is a second-order tensor and is expressed in a matrix form as

(8) κ=κ11κ12κ31 κ22κ23sym. κ33.

The thermal conductivity tensor **κ** obeys the following law of transformation.

(9) $$\kappa^{\prime }=L\kappa L^{T}$$

where **L** is direction cosine expressed as [32].

(10) $$L=\left[\begin{array}{ccc} l_{1} & m_{1} & n_{1}\\ l_{2} & m_{2} & n_{2}\\ l_{3} & m_{3} & n_{3} \end{array}\right].$$

### 2.2. Microstructure of Materials

**Figure 2:**
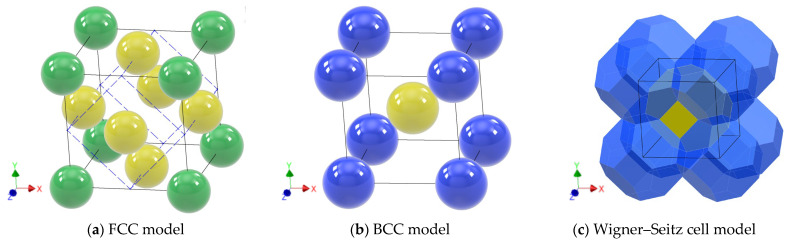
Microstructures of FCC, BCC and Wigner–Seitz cell models.

This study focuses on cases where inclusions are equally dispersed in matrices to eliminate the effect of randomness on the macroscopic characteristics of the materials. In these cases, FCC (face-centered cubic) and BCC (body-centered cubic) can be considered as candidates of microstructures. Spherical fillers with a diameter of 50 μm in FCC and BCC configurations were analyzed in this study as shown in Figure 2a,b. In addition, inclusion with a polyhedral shape which can realize 100% of packing, known as the Wigner–Seitz cell [33], was introduced as shown in Figure 2c. Inclusions come to contact when their volumetric fractions are at the maximum for each configuration of the inclusions. The maximum volumetric fraction for the FCC model is 74% because the unit cell of the FCC model includes eight portions of one eighth and six portions of half a spheric inclusion, and the ratio of their volume to the total volume of the unit cell is 74%. In the same way, the maximum volume fraction for the BCC model is calculated to be 68%. In the Wigner–Seitz cell model, inclusions come to contact only when their volumetric fraction is 100%. With the Wigner–Seitz cell, a very high volumetric fraction can be achieved without contact by placing the inclusions with some small clearances.

**Figure 3:**
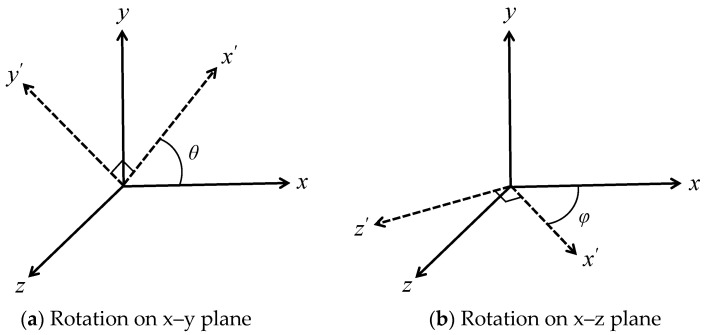
Transformation of Cartesian coordinates.

Cartesian coordinate systems shown in Figure 2a–c in which each axis corresponds to the directions of segments connecting inclusions at the corner of the unit cell are used in the analyses. With this basis, an arbitrary coordinate system can be configured via transformation with angles *θ*, *φ* defined in Figure 3. When *θ* = 45° in the FCC model, the closest pair of inclusions is aligned to the *x*’ axis as shown by the blue dashed line in Figure 2a. This configuration named transformed FCC was also analyzed to investigate anisotropy.

### 2.3. Finite Element Models

**Figure 4:**
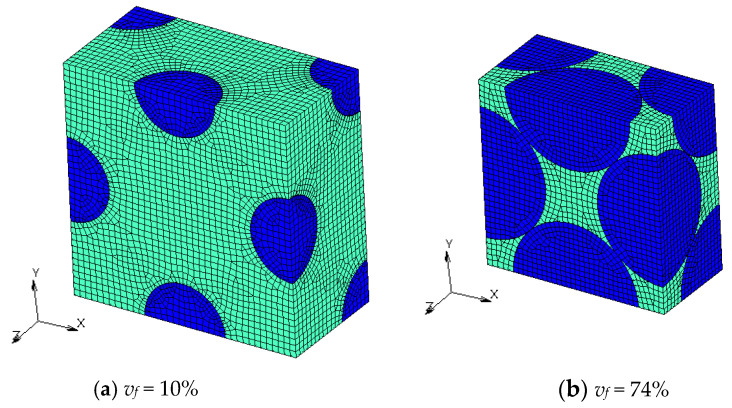
Numerical models of FCC microstructures with different volume fractions *v_f_*.

**Figure 5:**
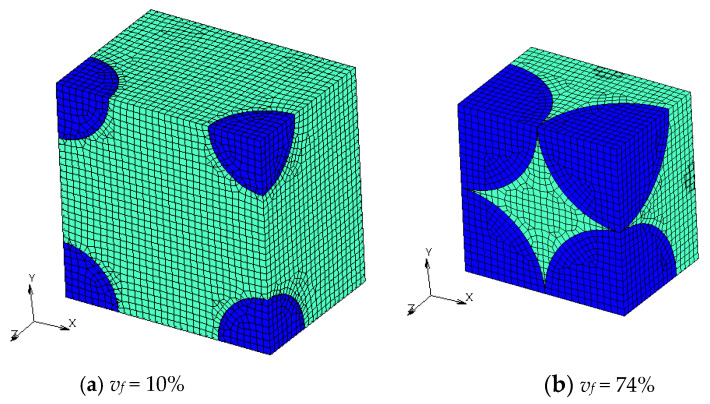
Numerical models of transformed FCC microstructures with different volume fractions.

**Figure 6:**
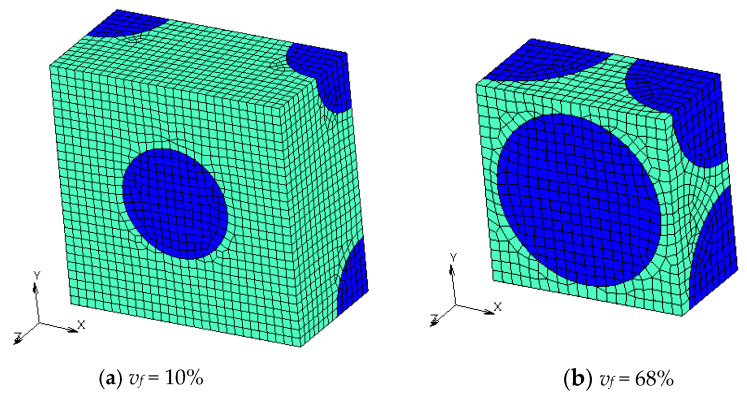
Numerical models of BCC microstructures with different volume fractions *v_f_*.

**Figure 7:**
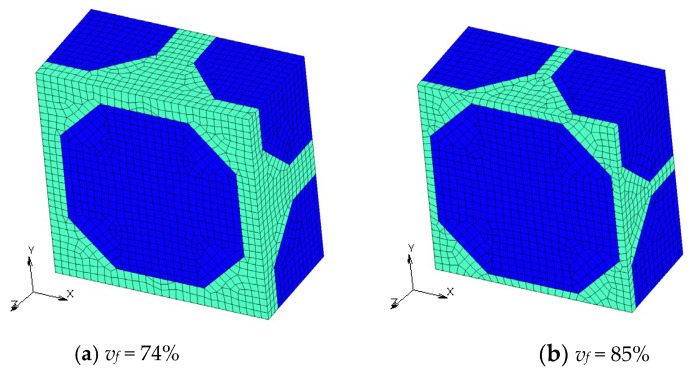
Numerical models of microstructures with Wigner-Seitz cells with different *v_f_*.

Finite element models with different *v_f_* are shown in Figure 4, Figure 5, Figure 6 and Figure 7. Regions with a dark blue color show inclusions and the one with a light green color shows the matrix. A half model according to plane symmetricity in the *z* axis in addition to the cyclic symmetricity in the *x* and *y* axes was created with low-order hexahedral elements.

Marc 2017 and Mentat 2017 (MSC Software Inc., Newport Beach, CA, USA) were used as solver and pre–post-processors, respectively. The Ryzen 7 5700G 8-core Processor (AMD Inc., Santa Clara, CA, USA) was used with CentOS 7 as a platform.

**Figure 8:**
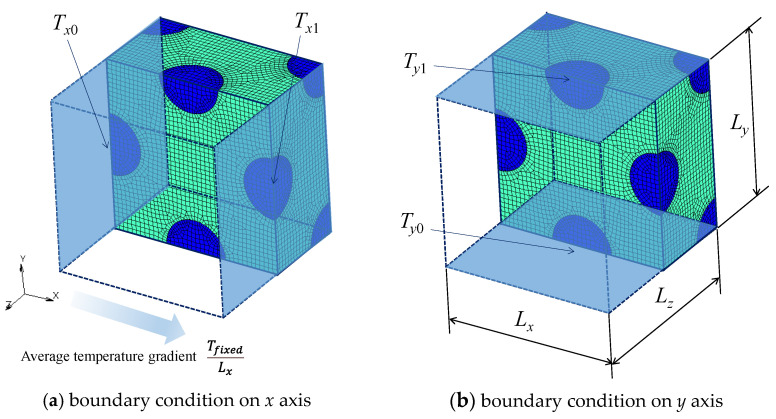
Boundary conditions of FEA models considering cyclic symmetricity.

As already mentioned, the boundary conditions considering cyclic symmetricity were defined as follows [14]: The average temperature gradient in the *x* direction was assumed to be constant, and no temperature gradients in the *y* and *z* axis were assumed. The average temperature gradient in the *x* axis was defined as shown in Figure 8a. MPC (multi-point constraint) capability of the finite element solver was used to define the following relationship [32].

(11) $$T_{x1}-T_{x0}=T_{fixed}$$

where *T_x_*_0_ is the nodal temperature at the symmetric plane on the left side and *T_x_*_1_ is the corresponding one on the right side. *T_fixed_* is the difference in temperature between the two symmetric planes. A representative node which connects the nodes on both symmetric surfaces was defined for the MPCs and the temperature of the representative node *T_fixed_* was set to 1 K in the present analyses. There were a lot of pairs of nodes at corresponding locations at the symmetric planes. Therefore, the relationship in Equation (11) was defined for each pair of these nodes using a common representative node. This condition defines the difference in temperature between two whole symmetric planes using the single representative node. In a similar manner to that shown in Figure 8b, MPCs for cyclic symmetricity in the *y* axis were defined using the following equation.

(12) $$T_{y1}=T_{y0}$$

where *T_y_*_0_ and *T_y_*_1_ are nodal temperatures at the bottom and top surfaces, respectively. The condition of plane symmetry in the *z* axis was automatically satisfied with no constraint of nodes on the plane of symmetry.

Thermal conduction at the interfaces between inclusion and the matrix was analyzed with the following equation using a capability of contact analysis in the finite element solver [32].

(13) $$q=H\left(T_{inclusion}-T_{matrix}\right)$$

where *H* is interfacial thermal conductivity which is reciprocal of interfacial thermal resistance *λ*. *T_inclusion_* and *T_matrix_* are temperatures at nodes on an interface of inclusions and the matrix, respectively.

Steady-state heat transfer analysis was conducted without consideration for the dependencies of material properties on other physical values such as time and temperature. Therefore, the solution did not show non-linearity, and the state of the thermal equilibrium under the applied boundary conditions was obtained without iteration.

Finite element mesh was generated in the following way so that the boundary conditions mentioned above were properly defined. First, all of the outer surfaces of bodies were meshed with quadrilateral elements. Mesh patterns on two planes of symmetry facing each other must be the same to define the MPCs, so the elements on one of the symmetry planes were duplicated on another side of the symmetry plane. This procedure was repeated for the *x* and *y* directions. Then, three-dimensional regions wrapped by quadrilateral elements on the surface were meshed with hexahedral elements.

The material properties used in the analyses are shown in Table 1. Polymer matrix composites with graphite fillers with a grain size of 50 μm were assumed in the present study. Synthetic graphite powder with a grain size from 1.0 to more than 100 μm is commercially available [1]. The thermal conductivity of graphite is more than 200 W/(m·K) depending on its carbon content [2]. Various types of polymers are adopted for the matrix of thermally conductive composites such as epoxy [1] and polyethylene [2,3]. The range of thermal conductivity of these polymers is from about 0.2 to 0.4 W/(m·K) [1,2]. In the present study, thermal conductivities for fillers and matrix were chosen to be 250 and 0.2 W/(m·K), respectively. The ratio of the thermal conductivity of the fillers to that of the matrix *K_f_* was 1250.

### 2.4. Evaluation of Effective Thermal Conductivities

Effective thermal conductivities of the composites on a macroscopic scale were evaluated from the results of the finite element analyses in the following way: the total amount of heat conduction *Q_react_* in the *x* axis can be obtained at the representative node of the MPCs defined by Equation (11). When the sizes of the unit cell models in *x*, *y* and *z* are *L_x_*, *L_y_* and *L_z_*, respectively, as shown in Figure 8b, the average heat flux in the *x* axis *q_x_* is given by

(14) $$q_{x}=\frac{Q_{react}}{L_{y}L_{z}}.$$

As the difference in temperature between two symmetric surfaces in the *x* axis is *T_fixed_*, the average temperature gradient in the *x* axis is expressed as

(15) $$\frac{dT}{dx}=\frac{T_{fixed}}{L_{x}}.$$

By substituting Equations (14) and (15) into Equation (5), the effective thermal conductivity of the composites is obtained as follows.

(16) $$\kappa_{11}=\frac{Q_{react}}{L_{y}L_{z}}\frac{L_{x}}{T_{fixed}}.$$

By solving the systems of Equations (5)–(9) with the results of FEA with different coordinate systems such as FCC and transformed FCC models with the consideration of symmetricity, a full matrix of thermal conductivity **κ** can be obtained.

## 3. Results and Discussions

### 3.1. Effect of Contact between Fillers

Table 2 shows the effective thermal conductivities calculated from the results of the FEA without consideration for thermal resistance based on Equation (16). The values from the FCC models and transformed FCC models are almost coincident in all cases of *v_f_*, which indicates that the materials are isotropic. Although the numerical calculation was conducted with real numbers that have 15 digits, the results were shown in 4 significant digits.

**Figure 9:**
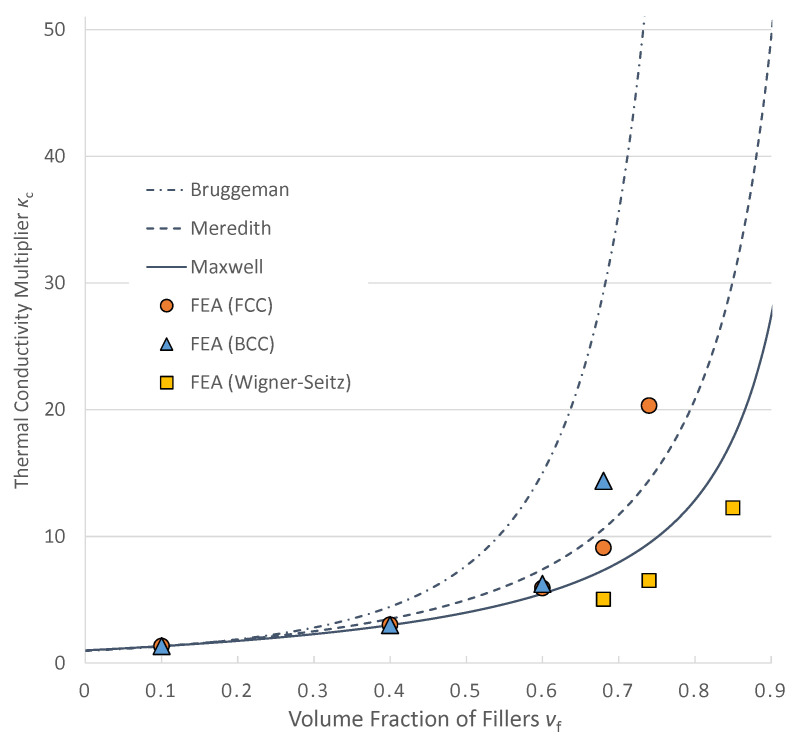
Comparison between results from FEA and theories.

A comparison between results obtained from the finite element analyses and theoretical equations is shown in Figure 9. The horizontal axis indicates the volume fraction of fillers *v_f_* and the vertical axis indicates the ratio of thermal conductivities of composites to that of the matrix *K_c_*. The value on the vertical axis can be interpreted as how much the effective thermal conductivities are improved by compounding the fillers. For example, the value on the horizontal axis becomes 2 when the effective thermal conductivity of the composite is double that of the polymer without fillers. Results from the FCC and BCC models agreed with Maxwell’s equation when *v_f_* = 10 and 40%. These results with low *v_f_* are rational according to the fact that Maxwell’s formulation does not take interaction between fillers into account. When *v_f_* = 60%, they have a value between Maxwell’s theory and Meredith’s theory. Bruggeman’s theory, which tends to overestimate the local interaction, has a higher value. When *v_f_* = 68%, which is the maximum packing fraction for the BCC model, the effective conductivity is almost double that of the FCC model with the same volume fraction. With this same volume fraction, contact occurs in the BCC model but does not occur in the FCC model. Similarly, the value of the FCC model with the maximum packing fraction of *v_f_* = 74% is more than double the value of the Wigner–Seitz cell model in which contact occurs only when *v_f_* = 100%. In this case, the most significant difference is the existence of contact, too. These results indicate that contact has a significant effect on an improvement in thermal conductivity of the composites.

It should be noted that the thermal conductivities with a high volumetric fraction of fillers are not necessarily higher than those with a lower volumetric fraction. All Wigner–Seitz cell models show lower conductivity than that of other models with higher volumetric fraction, i.e., the Wigner–Seitz cell model with 85% of volume fraction shows about half of the thermal conductivity of the FCC model with 74% of volumetric fraction. Moreover, the effective thermal conductivities of Wigner–Seitz cell models are the smallest in all of the cases with the same *v_f_*. Because the shape of Wigner–Seitz cells is determined so that neighboring cells completely fill the empty space, by placing the cells with some clearances, the highest volume fraction without contact can be realized. Therefore, distance between the fillers in the models with a Wigner–Seitz cell is supposed to be larger than that with any other shapes of fillers when the volume fractions are the same. Thus, the effective thermal conductivities of Wigner–Seitz cell models can be considered to be lower bound for composites with equally dispersed fillers.

**Figure 10:**
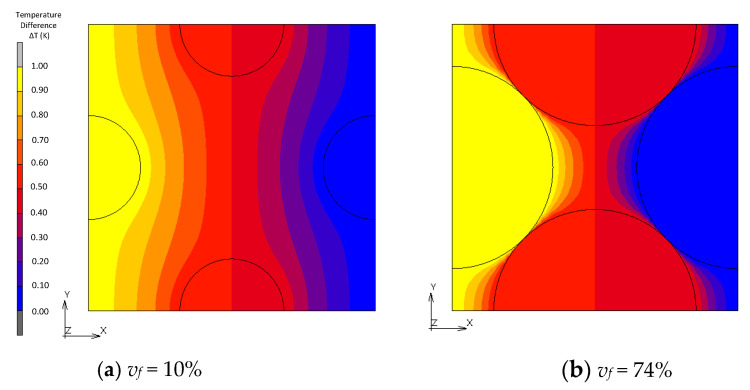
Temperature distribution of FCC models.

**Figure 11:**
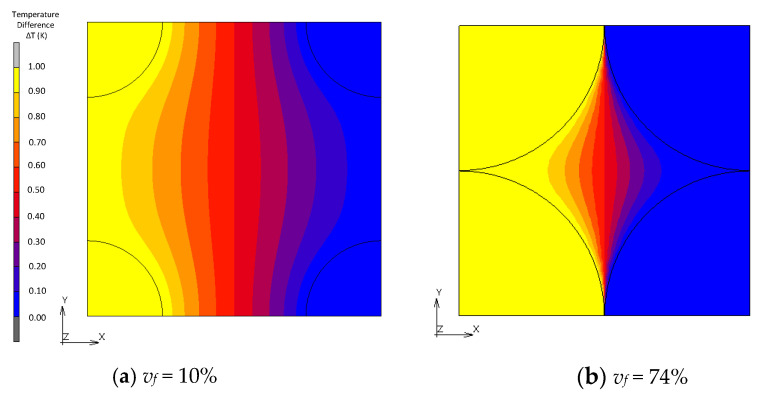
Temperature distribution of transformed FCC models.

**Figure 12:**
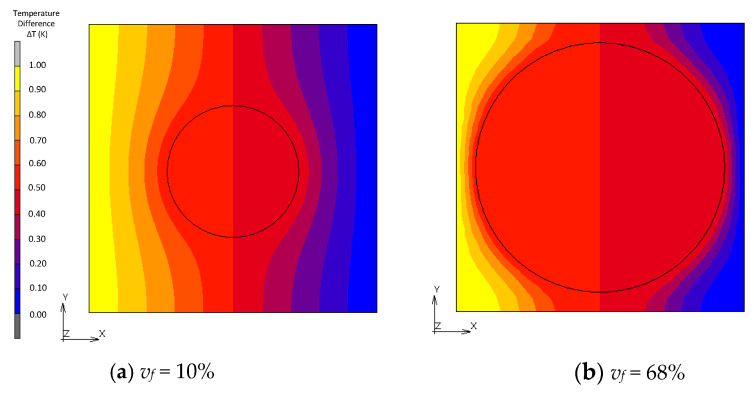
Temperature distribution of BCC models.

**Figure 13:**
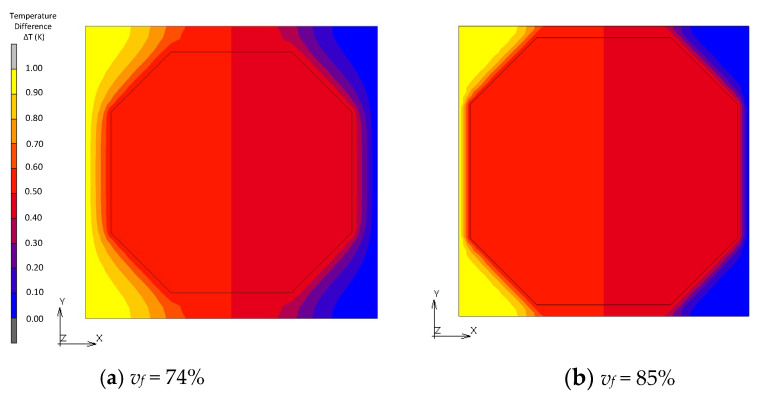
Temperature distribution of Wigner–Seitz cell models.

Figure 10, Figure 11, Figure 12 and Figure 13 show a distribution of temperature in FCC, transformed FCC, BCC and Wigner–Seitz cell models with different *v_f_*. In the results of FEA with *v_f_* as low as 10%, temperature is gradually changed in the matrix whose thermal conductivity is low, and temperature in the fillers with high thermal conductivity is almost constant. In Maxwell’s theory, it is assumed that a single filler is located at the center of the unit cell and that temperature at the location of the filler gradually converges to a constant at infinity. The temperature distribution of the FEA agrees with this assumption. This explains the coincidence of the effective thermal conductivities obtained from the FEA and Maxwell’s theory when *v_f_* is low. In the cases with a maximum packed fraction such as the BCC model with *v_f_* = 68% and FCC model with *v_f_* = 68%, the fillers came to contact, and the temperature at the point of contact looked almost discontinuous due to an intense temperature change. This means that there is an abrupt temperature gradient at the point of contact. This behavior explains the difference between the results from FEA and theories. There is a gradual temperature gradient in the matrix even when contact occurs. This indicates that the matrix transfers moderate heat flux according to Fourier’s law and does not have a role as a main thermal path. In the Wigner–Seitz cell model, a gradual temperature gradient is observed even with *v_f_* as high as 68% and 74% because there is no contact point to directly transfer heat flux between fillers. This explains the low thermal conductivity of these models.

**Figure 14:**
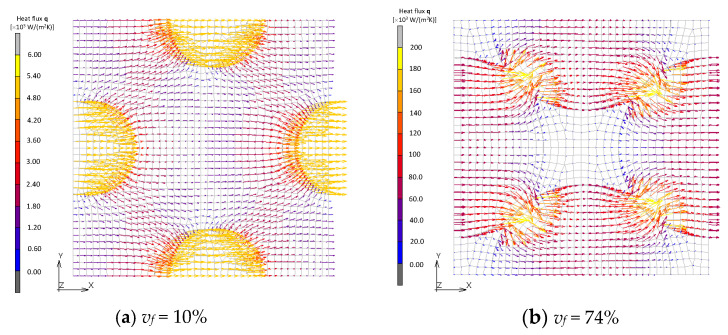
Heat flux field of FCC models.

**Figure 15:**
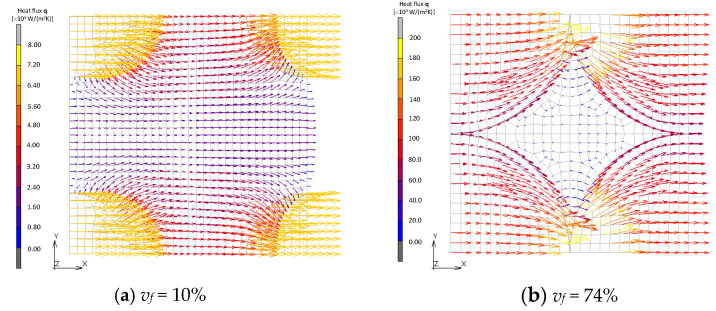
Heat flux field of transformed FCC models.

The distribution of the heat flux is discussed next. Figure 14 and Figure 15 show vector fields of the heat flux in the FCC and transformed FCC models. Distributions at the plane of symmetry in the *z* axis are shown. In the case where *v_f_* = 10%, the flux radially emanates from a filler and reaches neighboring fillers passing through the matrix. In the cases where *v_f_* = 68%, on the other hand, the flux from a filler directly transfers to the neighboring filler and little flux transfers through the matrix. It is observed that, under the same average temperature gradient for the two models, the amount of heat flux significantly increases due to a thermal path being formed by the contact.

**Figure 16:**
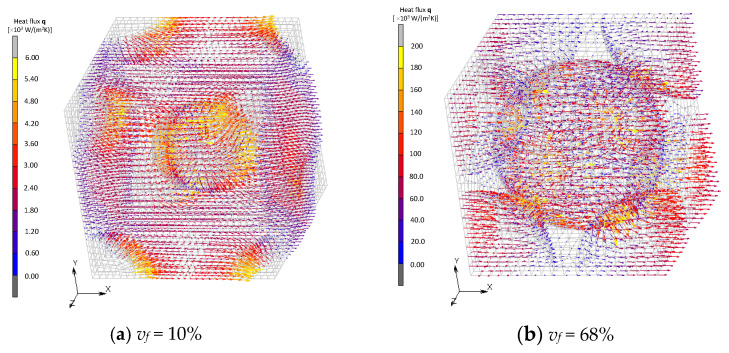
Heat flux field of BCC models.

**Figure 17:**
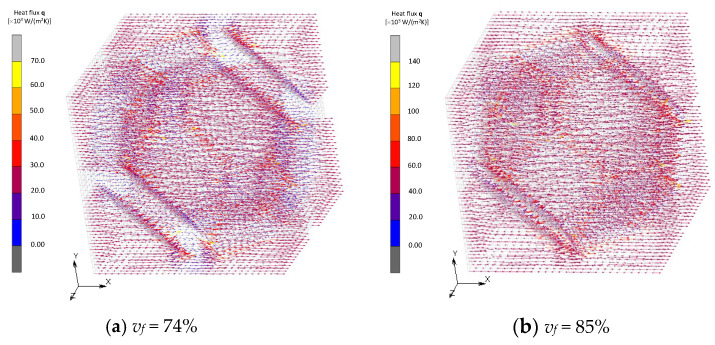
Heat flux field of Wigner–Seitz cell models.

Vector fields of the heat flux in BCC and Wigner–Seitz cell models are shown in Figure 16 and Figure 17. Direct heat conduction from one filler to another at the point of contact is dominant in the model of BCC, which is similar to the FCC model when its volume fraction is maximum. On the other hand, the Wigner–Seitz cell model with the same volume fraction exhibits moderate heat flux between fillers due to the non-existence of contact points. These results show that the contact is the dominant factor for an improvement in effective thermal conductivity. The formation of the thermal path, i.e., percolation, does not necessarily occur with a high volume fraction of fillers because the occurrence of contact between fillers depends not only on the fraction but also the shape and configuration of the fillers.

### 3.2. Effect of Interfacial Thermal Resistance

**Figure 18:**
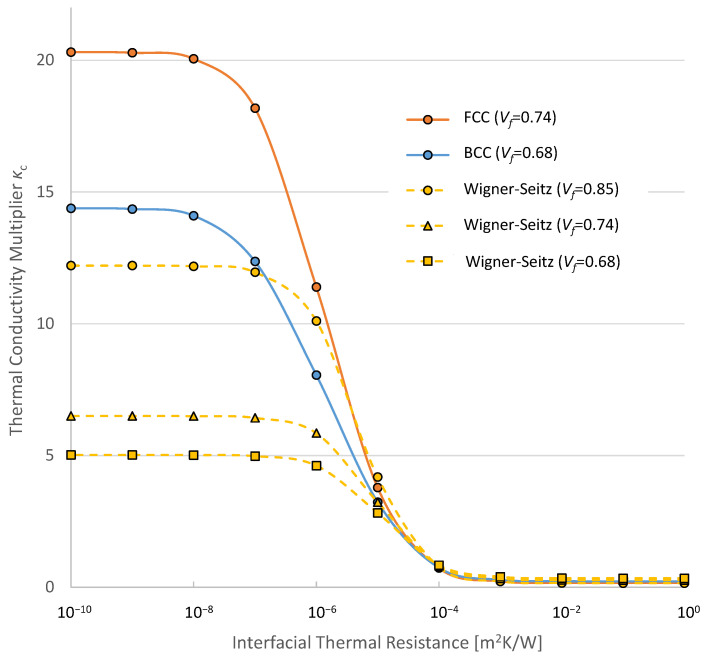
Effective thermal conductivities with different interfacial thermal resistances.

Figure 18 shows the relationships between an improvement in effective thermal conductivity and interfacial thermal resistance. The horizontal axis indicates interfacial thermal resistance on the logarithmic scale and the vertical axis indicates the ratio of thermal conductivities of composites to that of matrix *K_c_*.

The theoretical value of interfacial thermal resistance *λ* is considered as the value when the perfect interface between two materials is obtained. The theoretical value based on the phonon diffuse mismatch model is high when two materials that compose the interface are dissimilar. For instance, the interface between lead and diamond has interfacial thermal resistance in the order of 10^−7^ m^2^ K/W. On the other hand, the interface composed of two diamonds has an order of 10^−10^ [30]. In Figure 18, the difference in the effective thermal conductivities between FCC models with *λ* = 10^−8^ and *λ* = 10^−7^ m^2^ K/W is about 10%. This means that the thermal resistance of the interface composed of dissimilar materials has considerable influence on the effective thermal conductivity even when the surface treatment of the interface is perfectly conducted. The interface between graphite and polymers that the present analyses adopted is one of the examples of this kind.

It is reported that interfacial thermal resistance becomes as high as the order of 10^−4^ m^2^ K/W in practice when surface roughness of the interface is large [24]. In Figure 18, effective thermal conductivities significantly change in a range of interfacial thermal resistance *λ* from 10^−7^ to 10^−4^ m^2^ K/W in all cases. This indicates that, even when sufficient thermal paths exist due to the contact between fillers as shown in Section 3.1, heat transfer at the interface is greatly hindered by interfacial thermal resistance. Therefore, a good treatment on the interface between the matrix and fillers is quite important to obtain high thermal conductivity of composites. The interfacial thermal resistance being as high as 10^−4^ m^2^ K/W is due to the rough interface mentioned above which almost eliminated an improvement in the effective conductivity by the compounding of fillers.

## 4. Study Limitations

In the analyses in the present study with the assumption that the fillers were equally dispersed, the fillers came to contact only when the fraction of the fillers was the maximum for their configurations. Further study is going to be conducted on the interaction between fillers with consideration for randomness in the configuration of fillers.

## 5. Conclusions

Mechanisms for an improvement in effective thermal conductivity of composites were studied using a series of finite element analyses. Analyses of various microstructures with equally dispersed fillers with spherical and polyhedral shapes in configurations including FCC and BCC were conducted with consideration for local interaction such as contact between fillers and interfacial thermal resistance. The results were compared to theories in which the thermal local interactions were approximately considered. The influence of the local interaction on an improvement in effective thermal conductivity of composites was clarified using detailed analyses of temperature and heat flux field. The following conclusions were obtained from the analyses:

- Contact between fillers depending on the shape and configuration of the fillers had a more significant influence on the improvement in the effective thermal conductivity than the influence of an increase in volumetric fraction. The contact demonstrated the improvement that made the effective thermal conductivity more than double.
- The effective thermal conductivity decreased by approximately 10% due to interfacial thermal resistance in the order of 10^−7^ m^2^ K/W which was a realistic value for the interface between dissimilar materials even when a surface treatment of the interface was perfect.
- Interfacial thermal resistance that was higher than 10^−7^ m^2^ K/W had a significant influence on effective thermal conductivity. Interfacial thermal resistance must be lower than 10^−4^ m^2^ K/W to gain an improvement in the effective conductivity due to the compounding of fillers.

The present results showed an achievable range of effective thermal conductivity of dispersed composites and requirements of practical fabrication in the industries, not only about volume fraction of fillers but also about the treatment of interfaces between constituents.

## Figures and Tables

**Table 1:** Material properties used for FEA.

Properties	Fillers	Matrix
Thermal conductivity [W/(m·K)]	250	0.2
Diameter [μm]	50	-
Volume fraction [%]	BCC: 10, 40, 60, 68 ^1^ FCC: 10, 40, 60, 68, 74 ^1^ W-S Cell: 68, 74, 85	

^1^ Maximum packing fraction of each configuration.

**Table 2:** Thermal conductivities of composites obtained from the FEA.

Volume Fraction [%]	Effective Thermal Conductivities [w/(m·K)]			
	FCC	Transformed FCC	BCC	Wigner–Seitz Cell
10	1.331	1.330	1.328	-
40	3.009	2.999	2.996	-
60	5.915	5.876	6.245	-
68	-	-	14.38 ^1^	5.032
74	20.31 ^1^	20.67 ^1^	-	6.501
85	-	-	-	12.24

^1^ Results with maximum packing fraction.

## Data Availability

Not applicable.

## Nomenclature

*K_c_*	Ratio of thermal conductivity of the composites to that of the matrix material
*K_f_*	Ratio of thermal conductivity of fillers to that of the matrix material
*v_f_*	Volume fraction of fillers
*w*	Shape parameter for meredith’s model
**q**	Vector form of heat flux
**κ**	Matrix form of thermal conductivity
*T*	Temperature
*L*	Size of unit cell model
*θ*, *φ*	Angles in base Cartesian coordinate system
*λ*	Interfacial thermal resistance
*H*	Interfacial thermal conductivity
Subscripts	
*x*0, *x*1	Reference values on x axis
*y*0, *y*1	Reference values on y axis

## References

[1] Fu Y.X., He Z.X., Mo D.C., Lu S.S. (2014). Thermal conductivity enhancement with different fillers for epoxy resin adhesives. Appl. Therm. Eng..

[2] Ye C.M., Shentu B.Q., Weng Z.X. (2006). Thermal conductivity of high density polyethylene filled with graphite. J. Appl. Polym. Sci..

[3] He L., Ye Z., Zeng J., Yang X., Li D., Yang X., Chen Y., Huang Y. (2022). Enhancement in electrical and thermal properties of LDPE with Al_2_O_3_ and h-BN as nanofiller. Materials.

[4] Zain-Ul-Abdein M., Raza K., Khalid F.A., Mabrouki T. (2015). Numerical investigation of the effect of interfacial thermal resistance upon the thermal conductivity of copper/diamond composites. Mater. Des..

[5] Koosha N., Karimi-Sabet J., Moosavinn M.A., Amini Y. (2021). Improvement of synthesized graphene structure through various solvent liquids at low temperatures by chemical vapor deposition method. Mater. Sci. Eng. B.

[6] Burger N., Laachachi A., Ferriol M., Lutz M., Toniazzo V., Ruch D. (2016). Review of thermal conductivity in composites: Mechanisms, parameters and theory. Progr. Polym. Sci..

[7] Maxwell J.C. (1904). A Treatise on Electricity and Magnetism.

[8] Bruggeman D.A.G. (1935). Berechnung verschiedener physikalischer Konstanten von heterogenen Substanzen. I. Dielektrizitätskonstanten und Leitfähigkeiten der Mischkörper aus isotropen Substanzen. Ann. Phys..

[9] Meredith R.E. (1959). Studies on the Conductivities of Dispersions. Ph.D. Thesis.

[10] Bahrami M., Yovanovich M.M., Culham J.R. (2006). Effective thermal conductivity of rough spherical packed beds. Int. J. Heat Mass Transf..

[11] Kortschot M.T., Woodhams R.T. (1988). Computer simulation of the electrical conductivity of polymer composites containing metallic fillers. Polym. Compos..

[12] Ramani K., Vaidyanathan A. (1995). Finite element analysis of effective thermal conductivity of filled polymeric composites. J. Compos. Mater..

[13] Buonanno G., Carotenuto A. (2000). The effective thermal conductivity of packed beds of spheres for a finite contact area. Numer. Heat Transf. A Appl..

[14] Matt C.F., Cruz M.E. (2007). Effective thermal conductivity of composite materials with 3-D microstructures and interfacial thermal resistance. Numer. Heat Transf. A Appl..

[15] Haddadi M., Agoudjil B., Boudenne A., Garnier B. (2013). Analytical and numerical investigation on effective thermal conductivity of polymer composites filled with conductive hollow particle. Int. J. Thermophys..

[16] Pietrak K., Wisniewski T.S. (2015). A review of models for effective thermal conductivity of composite materials. J. Pow. Technol..

[17] Yvonnet J., He Q.C., Toulemonde C. (2008). Numerical modelling of the effective conductivities of composites with arbitrarily shaped inclusions and highly conducting interface. Compos. Sci. Technol..

[18] Muliana A.H., Kim J.S. (2010). A two-scale homogenization framework for nonlinear effective thermal conductivity of laminated composites. Acta Mech..

[19] Moumen A.E., Kanit T., Imad A., Minor H.E. (2015). Computational thermal conductivity in porous materials using homogenization techniques: Numerical and statistical approaches. Comput. Mater. Sci..

[20] Fang W., Gou J., Zhang H., Kang Q., Tao W.Q. (2016). Numerical predictions of the effective thermal conductivity for needled C/C-SiC composite materials. Numer. Heat Transf. A Appl..

[21] Koos G.L., Every A.G., Northrop G.A., Wolfe J.P. (1983). Critical-cone channeling of thermal phonons at a sapphire-metal interface. Phys. Rev. Lett..

[22] Eddison C.G., Wybourne M.N. (1985). Acoustic phonon scattering at sapphire surfaces coated with epitaxial silicon. J. Phys. C Solid State Phys..

[23] Swartz E.T., Pohl R.O. (1989). Thermal boundary resistance. Rev. Mod. Phys..

[24] Bhatt H., Donaldson K.Y., Hasselman D.P.H. (1990). Role of the interfacial thermal barrier in the effective thermal diffusivity/conductivity of SiC-fiber-reinforced reaction-bonded silicon nitride. J. Am. Ceram. Soc..

[25] Prasher R. (2006). Thermal interface materials: Historical perspective, status, and future directions. Proc. IEEE.

[26] Kapitza P.L. (1941). The study of heat transfer in helium II. J. Exp. Theor. Phys..

[27] Weis O. (1972). The solid-solid interface in thermal phonon radiation. J. Phys. Colloq..

[28] Khalatnikov I.M. (1959). Theory of diffusion and thermal conductvity for dilute solutions of He^3^ in helium II. J. Exp. Theor. Phys..

[29] Swartz E.T., Pohl R.O. (1987). Thermal boundary resistance from 0.5–300K. Phonon Scattering in Condensed Matter V.

[30] Wang H., Xu Y., Shimono M., Tanaka Y., Yamazaki M. (2007). Computation of Interfacial thermal resistance by phonon diffuse mismatch model. Mater. Trans..

[31] Landauer R. (1978). Electrical transport and optical properties of inhomogeneous media. Proc. Amer. Inst. Phys. Conf..

[32] Reddy J.N., Gartling D.K. (2020). The Finite Element Method in Heat Transfer and Fluid Dynamics.

[33] Kettle S.F.A., Norrby L.J. (1994). The Wigner-Seitz unit cell. J. Chem. Educ..

